# Monthly Summary

**Published:** 1888-03

**Authors:** 


### Monthly Summary.

---

The Application of Herbst's Method to Tin Gold Fillings.—*By Wm. M. Gabriel, M. R. C. S., L. D. S. Eng.*—When experimenting with tin in conjunction with gold, I filled one or two compound mesial cavities in bicuspid and molar teeth out of the mouth. These cavities I partially plugged with the combination metal and contoured the crowns with gold. During my work I was struck with the much longer time needed for the insertion of the combined foils when filling with a matrix, some form of which is often indispensable when dealing with distal cavities in bicuspids and molars. The much greater care necessary to secure good edges I could not help also noticing.

The fact that it was possible by Herbst's method to weld and make tin quite solid, suggested to me that one might be able to work the combined foils in this way, and that the gold might be burnished so as to adhere to the combination metal.

I find the combined metals quite as easy to work by Herbst's method as tin or gold separately, and no more difficulty in making an *un-annealed* gold cylinder adhere to a foundation of tin-gold, by means of a burnisher rotated in the engine, than to a mass of gold. I think, however, that no more reliance should be placed upon this union than upon so-called retaining points; but that the coronel half of the cavity should be shaped so as to make the gold self-retaining.

Personally, I prefer to burnish gold over all the tin-gold exposed, and then complete the operation with the mallet; but this, of course, is quite a matter of taste.

The matrices I use are a modification of Dr. Guilford's bands, retained in position by his clamps A, B, C and sometimes D. These bands I hope to further improve and speak of at some future time.

It is difficult to keep the ends of the bands now sold from getting disengaged from the little points on the clamps. The fact that they rarely fit the tooth to be filled is another disadvantage. This can often be overcome, if the band be too large, by a wooden wedge driven between the band and the side of the crown of the tooth. I prefer, however, whenever possible, to cut out the cavity, take an impression, and wedge at the first visit, fit a band to the model and by means of this fill at the next.

I use a sheet of tin foil (S. S. W. No. 6), laid between two sheets of *cohesive* gold foil (Keasing No. 4). This is cut into two or three strips; made into ropes and cut into pellets. The gold cylinders used are S. S. W. Velvet (small sizes).—*Dental Record.*

---

DARK JOINTS.—One other pest of the laboratory is dark joints, and there are various causes that creep in to render the cases we often wish to be our best anything but what we could desire. First of all, the great cause is not rubber, but *wax.* You may grind your joints ever so carefully, and boil them or rinse them out with hot water ever so thoroughly, but if there is a particle of wax in them, or if a particle has been in, you may look for discoloration. Take a piece of glass or porcelain, and melt some wax on its polished surface, and boil it in water ever so long, and as long as it is not rubbed it will remain greasy, and the heat of vulcanizing is sufficient to discolor it. Now a ground surface, like that of a fitted section, is far more difficult to clean than a polished surface; then too, hot wax is a solvent of rubber, and will lead the vulcanite into the joint. How can this be avoided? By simply keeping the wax out by keeping the ends of the sections wet. If wax gets on them, grind it off, for you cannot clean it.

One other cause. I noticed a brother in the profession, the other day, grinding up a case, and in order to see what kind o' a joint he was making he touched the sections with the tip of his tongue, thus wetting the joints. If any saliva gets into a joint before vulcanizing, there will be discoloration, as the organic matter contained in it will char and blacken under heat.—*Dr. J. H. Beebe, in Odontographic Journal.*

PERSEVERANCE AN IMPORTANT FACTOR.—In any line of business, the man who uses reasonable economy, and has the ability to give fair management and the perseverance to hold on, will, in a great majority of cases, make a success; while, on the other hand, the one who rushes into whatever he has undertaken with a spasmodic endeavor to win all at once, as a general rule wastes his energies, and often fails for sheer want of perseverance. The editor of the *Industrial Gazette* has observed that the man who starts in to do a day's work, and attempts to do as much in one hour as ought to be done in two, will usually find it necessary in a short time to take a rest, and while he is resting will lose valuable time, which he evidently feels he ought to make up, judging from the spasmodic efforts he will make when he starts into work again. But at night the man who works steady, but perseveringly, will be found to have accomplished the most, while usually he will also be found in a much better condition to commence again the next day.

So it is in business. One will seem to hustle around and make a considerable to do over what he is doing, and after wasting his energies in accomplishing what, by taking a little more time, could be done with very little effort, and then, because, as he thinks, he fails to meet the success he imagines he should, becomes discouraged, and is ready to make a change to something else. This, in a majority of cases, proves a loss, and, in consequence, he does not succeed as the energy he displays would seem to warrant. Another man, while he may not make a great display of his energies at the start, will go to work more systematicly, and will have better opportunities to economize, and in many cases to manage better than when he attempts to rush things. If he will but observe, he will be ready to take advantage of any favorable circumstances that may arise. The man who is constantly shifting about is always making a change at the wrong time, when a little perseverance would have brought him through all right. In all lines of business there are fluctuations, ups and downs, and to succeed we must persevere. It is when the odds seem against us that it seems the most important to persevere.—*Scientific American.*

THOROUGH EXAMINATION.—*By Dr. Elbridge C. Leach, Boston, Mass.*—Can anything too forcible be said on this point? Can there be anything more humiliating to the dentist than to look the patient's mouth over and then have the patient point out the one trouble for which he visited you? Can the dentist do better than to spend ample time on his examinations; pointing out the work which should be done at present and take good care to call the patient's attention to whatever work the dentist may discover which does not require immediate care? For instance, how often do we discover imperfections which do not require attention at once, like superficial decay on the neck of a tooth which it would be unwise to fill at the time of discovery, making a strong point of calling your patient's attention to it and to the fact that it does not require attention at once in the way of filling? How often do patients return after the completion of a course of operations and point out an apparent oversight of yours and thus imply your inefficiency in examination?—*The Archives of Dentistry.*

---

A VOICE RAISED AGAINST CARPETS.—A leading New York City physician distinguishes himself from his fellow-mortals by inveighing against carpets. He denounces them as ugly in appearance, and worst of all, as unclean and unhealthful. He says: "The carpet holds the poisons of all diseases, such as scarlet fever, diphtheria, and the like, long after the rest of the room is disinfected. I always remove the rug, even from a sick room, where I can find a rug to remove. The carpet retains dust and dirt, and the most careful housewife cannot keep it clean. It is impossible. Now, the rug may be taken up and thoroughly swept on both sides whenever the housekeeper wants to do so. Then there is nothing in the way of house furnishing so handsome as a painted and highly polished floor, decorated with Persian or Turkish rugs. No carpet ever made is so pleasing to the eye. Yes, the carpet must go, and I only wonder that the crusade against it has been delayed as long as it has."—*Pacific Record of Medicine and Pharmacy.*

EXPERIMEMTS.—As experiments I have inhaled chloroform, ether, bichloride of methylene, also nitrous oxide gas, always without ill-effects and receiving at times useful practical lessons.

My last test upon myself was with cocaine, when, about four months since, in the presence of my wife and some friends, I injected, slowly; twenty minims of the solution of cocaine, in close proximity to the thumb nail of my left hand, the result being utter insensibility of the part extending to the first joint. The non-sensitiveness continued for about an hour or more, during which period I subjected my thumb to most severe treatment, but without experiencing the slightest pain or even feeling; partial numbness existed for nearly two days, but unattended with any constitutional disturbance.

I am gradually losing my thumb-nail through the mechanical injury I perhaps stupidly inflicted.

My experience thus far is sufficiently inspiriting to make me believe that cocaine is a most valuable drug, and could, I feel convinced, be used with advantage in minor and even major surgical operations, often preventing the infliction of unnecessary pain.—*A. W. Furber, L. D. S., in British Journal of Dental Science.*

---

WHAT IS PAIN?—It is one thing to know a thing, it is quite another thing to tell it. A definition for pain seems to have been more than lexicographers could "masticate." The *Popular Science News* says:

"An eminent physiologist calls it 'an excess of the sense of touch,' and another 'hyperæsthesia of the sensory fibres.' Dunglison, in his Medical Dictionary, says 'it is a disagreeable sensation, which scarcely admits of definition;' and Gardner, in his work of the same title, under 'pain,' says 'see Dolor,' and turning to 'Dolor,' we find it concisely explained as 'pain.' The great French Dictionnaire des Science Medicales coolly and conveniently tells us that to define it is superfluous. Professor Erb, in Ziemssen's Cyclopædia, after some discussion, comes to the conclusion 'that pain is a new sensation, experienced when excitation of the nerves reaches a certain intensity.'

Perhaps the Imperial Dictionary covers the ground in describing it as 'an uneasy sensation in animal bodies, of any degree from slight uneasiness to extreme distress or torture, proceeding from pressure, tension or spasm, separation of parts by violence, or any derangement of functions.' This is also Webster's definition, and is really not more exact than some of the others, although longer."—*Chicago Medical Times.*

---

GOLD IS AND WILL REMAIN THE KING OF FILLING-MATERIALS, and the practitioner of the most mediocre ability is using it more and more. As he learns to preserve more of the teeth with the plastics which he formerly extracted, so now he is also ambitious to extend his knowledge of gold filling and increase his practice of it. When the most ordinary dentist is ambitious to do fine work, and that ambition is fix habit, he is a saved man. By persistent study and the practice of better methods, he will soon be lifted above mediocrity, and his further progress is then assured. Just as sure as he is desirous of progressing, will he develop and improve. No man is so poor a workman, or so unpracticed a student, or so illiterate, but that, with a sincere desire to improve, he may attain a degree of ability that will be a pride to himself and friends; and in the use of gold for filling, no man is so unfortunate in early training but that, by proper study and application, he may attain a respectable ability.—*A. H. Thompson, Topeka.*

---

FLIES IN THE OPERATING ROOM.—Every one must have experienced the intolerable nuisance flies are during the summer in the waiting and operating rooms. In Switzerland oil of bays (huil de laurier) is used to prevent the visits of these "household friends." A coat of this oil is applied to the walls, which effectually excludes flies of all kinds. This remedy has also been tried and found effectually in the south of France in preserving gilt frames, chandeliers, etc., from becoming soiled. It is even remarked that the flies soon avoid the rooms where the application is employed.—*British Journal of Dental Science.*

ANCHOR PINS.—For some time I have been using the double headed pins used by the manufacturers of artificial teeth, as anchor pins in filling badly decayed and broken down teeth, and have come to regard them as valuable in saving such teeth.

If exposed, destroy the pulp and fill the root canal with a guttapercha cone. I follow this with filling the pulp cavity with oxyphosphate, and before it hardens I put in one or more of the pins so that their round heads will project into the cavity far enough above the surface of the cement to act as anchors for either a gold or amalgam facing, and pack the cement closely around the pins. I wait awhile and then remove the excess and clear the margin of the cavity before it gets hard.

When it has hardened sufficiently I made a shallow cavity with the heads of the pins projecting into it. Around them I anchor the gold or amalgam.

For incisors I use one large pin, and for bicuspids or molars, two or more pins slanting them so as to give them greater strength.

I have built up incisors, cuspids, bicuspids, and molars in this way where it seemed impossible to get retaining points sufficient to hold a filling, and had made good useful teeth of them.—*Dr. L. West, Marionville, Mo.*

---

THE CULTIVATION OF THE FACULTY OF KNOWING is of incomparably greater moment than the mere acquisition of knowledge. He is not the best of explorers or campaigners who is the most burdened with baggage, but he who knows how to forage well, and how to make the best possible use of what he has or can obtain. So it is with the student; to know how to learn, so that when need arises knowledge may be quickly obtained, is a better provision for the business of life than is afforded by the largest or richest store of information packed away in the memory—perhaps so packed as to be inaccessible when wanted. If students for themselves, and teachers for their pupils, would insist on the importance of "learning how to learn," instead of cramming, there would be fewer disappointments in life, and greater and more enduring successes. The vanity of carrying a huge quantity of information for the sake of display is contemptible. The folly of attaching any real value to vast stores of knowledge is pitiful. The only brain properly worth carrying about is the power of finding at pleasure and learning at will precisely what is wanted; and this power cannot be acquired without considerable practice in the art of learning—an art which the student should make it a first object of his best endeavor to master.—*Lancet.*

---

COCAINE AND CARBOLIC ACID ANÆSTHESIA.—M. Vian (*London Medical Record*) found that Witzel's method of injecting 1½ grains of cocaine in dental operations gave very good results, but two out of eight patients experienced constitutional disturbances of an alarming character, and several others were markedly inconvenienced. He therefore dissolved three fourths of a grain of cocaine in eight minims of a two per cent. solution of carbolic acid, which he used as follows: Eight minims of the above mixture are slowly injected, half on the labial and half on the lingual or palatal aspect of the gum at a spot situated half-way between the neck of the tooth and the presumed end of the root. A finger should be kept over the site of the puncture to prevent the escape of the fluid, and the surrounding parts should be protected by pledgets of cotton-wool. The patient rinses out his mouth with water, and five minutes are allowed to elapse before beginning the operation. M. Vian has operated on eighty-six patients, with a very successful result in all; no disturbance, except of an emotional kind, ever following. Very good results were obtained by the use of the carbolized solution alone, and he is disposed to consider carbolic acid as efficient a local anæsthetic as cocaine for dental purposes.—*The Odontographic Journal.*

---

TALENT VS. GENIUS.—Talent is full of thoughts; genius is full of thought. Talent has definite plans and activities; genius has power that is an unknown quantity, and aims quite as indefinite. Talent has many ends in view, and prides itself on the extent of its vision; the aim of genius is concentrated, and its vision is narrow. Talent is cool, logical, convincing; genius is hot-headed, jumps at conclusions, and is often the laughing-stock of observers. Talent gains its ends by degrees, occupies its vantage ground with dignity, and is the commander of its situation; genius worms itself to its positions stealthily, knows no dignity and cares for no praise. Talent is the plodding investigator, the exhaustive student and the orator of the rostrum; genius is the alchemist, the close observer and the secret, cunning worker. Talent investigates the reason of things, prides itself on the logic of its conclusions, and avers a thing cannot be that does not accord with the rules of science; genius looks for the results, not the reasons for it; knows no logic but facts, and doubts nothing. The world would go awry if it was not for talent; but its progress is largely due to genius; its results are sometimes wonderful, quite surprising the credulity of talent, but accepted by the common people with gratitude.

But talent may be sleeping, and genius may spend its strength on nonsense. To be of use both must have a worthy purpose, and each must be guided and inspired by a motive that shall raise it above the miasma of lust and selfishness, or either will become poisoned and diseased, a curse to the world.

We all have some talent and some genius; so little of either, perhaps, that great exertion must be made if we would rise into any respectability and success. But we can use what we have, and use it to the utmost; then we shall not be pigmies, though we may not be giants; there will be many of shorter stature, though there will be some taller, and we shall have the consciousness of seizing every opportunity for self-development, though many may run past us.—*Items of Interest.*

---

POISONING WITH IODOL.—Pallin (*Hygiea; Ctrlbl. f. Chir.*) gives an account of a case of necrosis of the clavicle in which an operation was performed for the removal of a sequestrum and 75 grains of iodol were applied to the wound. During the evening of the same day the patient became delirious, and on the following day his temperature was 102 2° F., his pulse was 136, small and irregular, and he vomited and was apathetic. The urine showed traces of albumen and a weak iodine reaction. Although the dressing was changed at once, all the iodol being washed out of the wound and bismuth applied in its place, the symptoms of poisoning lasted four days longer, and for a fortnight iodine was to be recognized in the urine.—*Ibid.*

---

IN REGARD TO OBTUNDENTS, a good deal depends upon the operator and his ability to influence the patient. In so many cases of sensitiveness as we have to deal with, we must have some obtundent. Absolute alcohol is good in some places. When the rubber has been adjusted and the cavity dry, pain is more pronounced upon application of any medicine, and especially if it be a cold liquid. It is better to apply the obtundent warm, as there is less pain, and it also increases the local anæsthetic property. Alcohol, or anything that evaporates the water in the tooth, causes pain for a moment, but this soon passes off and we get the desired effect.—*Dr. A. W. Harlan, in Ohio Journal.*

---

A RECENT writer inveighs against indiscriminate use of tea and coffee, especially in the young. He is of the opinion that against the practice of giving them to children we can not speak too strongly. Childhood is the period when the nervous activity is at its greatest. The brain is very busy in receiving new impressions. Reflex action, co-ordination of muscles, and the special senses are all under a constant course of training. The nervous system is pushed to its utmost capacity, and long is the list of victims that follow this overstimulation. In these little people, nothing but harm can come from the use of such cerebral stimulants as tea and coffee.—*Power and Transmission.*

---

In using steel jack screws the corrosive effect of the oral secretions may be prevented, and the screws nicely lubricated, by immersing them in hot paraffine. This should be done every three or four days.

